# Class II Phosphoinositide 3-Kinases Contribute to Endothelial Cells Morphogenesis

**DOI:** 10.1371/journal.pone.0053808

**Published:** 2013-01-08

**Authors:** Gianpaolo Tibolla, Roberto Piñeiro, Daniela Chiozzotto, Ioanna Mavrommati, Ann P. Wheeler, Giuseppe Danilo Norata, Alberico Luigi Catapano, Tania Maffucci, Marco Falasca

**Affiliations:** 1 Queen Mary University of London, Barts and The London School of Medicine and Dentistry, Blizard Institute, Centre for Diabetes, Inositide Signalling Group, London, United Kingdom; 2 Department of Pharmacological and Biomolecular Sciences, University of Milan, Milan, Italy; 3 Center for the Study of Atherosclerosis, Società Italiana Studio Aterosclerosi, Bassini Hospital, Cinisello Balsamo, Italy; 4 Queen Mary University of London, Barts and The London School of Medicine and Dentistry, Blizard Advanced Light Microscopy Core Facility, London, United Kingdom; 5 Istituto di Ricerca e Cura a Carattere Scientifico MultiMedica, Milan, Italy; UAE University, United Arab Emirates

## Abstract

The question of whether the distinct isoforms of the family of enzymes phosphoinositide 3-kinases (PI3Ks) play redundant roles within a cell or whether they control distinct cellular processes or distinct steps within the same cellular process has gained considerable importance in the recent years due to the development of inhibitors able to selectively target individual isoforms. It is important to understand whether inhibition of one PI3K can result in compensatory effect from other isoform(s) and therefore whether strategies aimed at simultaneously blocking more than one PI3K may be needed. In this study we investigated the relative contribution of distinct PI3K isoforms to endothelial cells (EC) functions specifically regulated by the sphingolipid sphingosine-1-phosphate (S1P) and by high density lipoproteins (HDL), the major carrier of S1P in human plasma. Here we show that a co-ordinated action of different PI3Ks is required to tightly regulate remodelling of EC on Matrigel, a process dependent on cell proliferation, apoptosis and migration. The contribution of each isoform to this process appears to be distinct, with the class II enzyme PI3K-C2β and the class IB isoform p110γ mainly regulating the S1P- and HDL-dependent EC migration and PI3K-C2α primarily controlling EC survival. Data further indicate that PI3K-C2β and p110γ control distinct steps involved in cell migration supporting the hypothesis that different PI3Ks regulate distinct cellular processes.

## Introduction

Since the identification of a phosphoinositide 3-kinase (PI3K) activity, several intracellular functions have been demonstrated to be dependent on this enzyme. Eight isoforms of PI3K exist in mammals, grouped into three classes according to their structure and substrate specificity [1], [2]. Class I PI3Ks, further divided into the subgroups IA and IB, are dimers composed of a catalytic and regulatory subunit. Class IA comprises the catalytic subunits p110α,β and δ whereas the only catalytic subunit of class IB PI3K is p110γ. Class I PI3Ks are mostly responsible for synthesis of the well established second messenger phosphatidylinositol 3,4,5-trisphosphate [PtdIns(3,4,5)*P_3_*] *in vivo*. Class II PI3Ks are monomers of high molecular weight [1], [2]. Three members exist, PI3K-C2α, β and γ and we demonstrated that they mainly catalyse the synthesis of phosphatidylinositol 3-phosphate (PtdIns3*P*) *in vivo* upon cellular stimulation [1], [3]–[6]. More recently PtdIns3*P* has been confirmed as the main specific product of PI3K-C2α by using MEFs upon induction of PI3K-C2α knockout [7]. Finally, the only class III PI3K isoform, hVps34, is mostly considered responsible for the synthesis of a constitutive pool of PtdIns3*P*.

The identification of different PI3K isoforms led to questioning whether these isoforms are redundant or rather they play distinct and not overlapping roles in cell signalling [1]. This question has become increasingly important, in particular with the development of isoform-specific PI3K inhibitors that require a clear understanding of distinct PI3K contribution in cellular biology and disease for the evaluation of their safety and efficacy and their pharmacological potential. Therefore it has become critical to define which isoform is important for regulation of which cellular function and to what extent [2]. Examples of co-operative action of distinct PI3Ks have been reported, further supporting the hypothesis that they do not play redundant functions [1], [9]. Among the eight PI3K isoforms, members of the class II subgroup remain the least investigated and there is still a lot that needs to be understood about the role of these isoforms [1], [6]. Since class II PI3Ks generate a different lipid product compared to class I [1], [3]–[7], it is likely that their roles are not overlapping with class I PI3Ks and therefore they may regulate distinct biological functions or may co-operate with class I PI3Ks in regulating some processes [1], [8], [9].

Sphingosine 1-phosphate (S1P) is generated by phosphorylation of sphingosine produced by the enzymatic hydrolysis of ceramide, a key intermediate in the synthesis of complex glycosylated sphingolipids, that can either be generated *de novo* or by breakdown of sphingomyelin through the activities of sphingomyelinase [10]. S1P levels in mammalian cells are regulated by the action of two sphingosine kinases, two phosphatases and a lyase [10]. Data have revealed that S1P can either act as a second messenger inside the cells or it can be secreted and it can bind specific G-protein coupled receptors (GPCR), the S1P receptors 1–5 [11]. Several cells are able to release S1P, although the major sources are platelets, erythrocytes and endothelial cells (EC). A gradient of S1P exists, with low levels in tissues and high levels in serum and plasma, where concentration of S1P is estimated to be around 400 and 200 nM respectively [12]. In plasma, S1P is found associated with albumin and lipoproteins, including low density lipoproteins, very low density lipoproteins and high density lipoproteins (HDL) [13]. The majority of the lipoprotein-bound S1P (∼54%) is associated with HDL, mostly concentrated in small dense subfraction HDL_3_, that show higher S1P/sphingomyelin ratio compared to large HDL_2_ [14]. S1P has a critical role in vascular development, in endothelial functions and regulation of angiogenesis in adults [15], [16]. Indeed, deletion of the S1P receptor 1 (EDG-1/S1P_1_) in mice is embryonic lethal because of massive haemorrhaging due to defect in vasculature stabilisation [17]. EC-specific deletion mimics the effect of the total knock out, indicating that S1P_1_ signalling in EC is critical for regulation of vascular maturation [18]. In addition, S1P has a key role in angiogenesis in adults and is able to stimulate EC proliferation, shape change and migration [19]. Furthermore there is growing evidence that S1P may account for many of the cardiovascular effects of HDL including the ability to promote vasodilation, EC migration and angiogenesis [20]. Specifically, S1P is one of the most potent inducers of chemotaxis in EC *in vitro* and of differentiation into capillary-like structures on Matrigel [21].

Data have indicated a key role for class I PI3K in S1P-dependent EC migration and it has been shown that at least two class I isoforms, the class IA p110β and the class IB p110γ, are involved in S1P-mediated cell migration in EC [22]. Not surprisingly, these are the isoforms able to be activated downstream of GPCR [2]. We recently demonstrated that at least some members of the class II subgroup of PI3Ks can also be activated downstream of GPCR activation. Specifically we showed that lysophosphatidic acid (LPA) can activate PI3K-C2β to regulate migration of cancer cells [5]. These data, together with results showing that the S1P-induced migration is partially resistant to treatment with the classical PI3K inhibitor wortmannin [22], a feature of at least some of the class II PI3K isoforms [1], [5], [6], [23], prompted us to investigate the potential involvement of class II PI3Ks on EC migration and capillary morphogenesis induced by S1P and HDL.

Here we show that downregulation of class II PI3Ks expression inhibits EC differentiation and morphogenesis. Detailed analysis of the specific role of these enzymes reveals that PI3K-C2β mainly regulates EC migration induced by treatment with either S1P or HDL_3_ whereas PI3K-C2α is involved in EC survival. Downregulation of p110γ also affects EC migration and our data indicate that PI3K-C2β and p110γ may have distinct, non overlapping role in regulation of this process. Taken together these data suggest a co-operative action of distinct PI3K isoforms to regulate EC morphogenesis.

## Materials and Methods

### Materials

Anti PI3K-C2α and anti PI3K-C2β were from BD Transduction Laboratories™ (BD Biosciences, Oxford, UK), anti Akt, anti ERK2, and anti actin from Santa Cruz Biotechnology (Santa Cruz, CA, USA), anti phospho Ser473 Akt, anti-phospho ERK1/2, anti S6 and anti- p110γ from Cell Signaling Technology (Danvers, MA, USA). Wortmannin, LY294002 and S1P were from Sigma Adlrich (St.Louis, MO, USA). AS252424 and AS605240 were from Alexis (Enzo® Life Sciences, Vinci, Italy). A66 was kindly provided by Prof Peter Shepherd (University of Auckland, New Zealand).

### Cell Culture and Downregulation of PI3K Isoforms Using siRNA

HUVEC were purchased from TCS CellWorks (Buckingham, UK) and grown in EGM™ Bulletkit™ or EGM™-2 Bulletkit™ (Lonza, Basel, Switzerland) supplemented with 10% FBS. siRNA duplexes used in this study were based on the human cDNA sequences encoding PI3K-C2α, PI3K-C2β and p110γ. Specific siRNAs targeting the class II isoforms PI3K-C2α (Sequence 1: 5′-AAGTCCAGTCACAGCGCAAAG-3′; Sequence 2: 5′-AAGTACAGAATGAGGAGATGG-3′) and PI3K-C2β (Sequence 1: 5′-AAGAATGCGACGCCTGGCAAG-3′) were custom-made by Qiagen (Qiagen, Milan, Italy). ON-TARGETplus SMARTpool siRNA (Sequence 1) and ON-TARGETplus single sequence (Sequence 2) targeting p110γ together with ON-TARGETplus single sequence targeting PI3K-C2β (Sequence 2) were from Dharmacon (Thermo Fisher Scientific, Inc., Waltham, MA, USA). Negative silencer non targeting siRNA (“scrambled”) was from Ambion® (Life Technologies Ltd, Paisley, UK). Transient transfections were performed using OligofectAMINE™ (Life Technologies Ltd, Paisley, UK) following the manufacturer instructions and efficiency of downregulation was evaluated by quantitative Real Time PCR and Western blotting.

### Lipoprotein Isolation

HDL subfraction 3 (HDL_3_ density 1.125–1.21 g/ml) was obtained from freshly isolated human plasma by preparative ultracentrifugation, dialysed versus PBS containing 0.01% EDTA and sterilised by filtration. Protein content was determined using the colorimetric Bradford assay. The Institutional Ethic Committee approved the study and informed written consent was obtained from all participating subjects. The study was conducted according to the standards of the Declaration of Helsinki and Good Clinical Practise.

### Real-time Quantitative (RT)-PCR

Total RNA was extracted and reverse transcribed as described [24]. Three µl of cDNA was amplified by real-time quantitative PCR with 1X SYBER Green universal PCR mastermix (Bio-Rad). The specificity of the Syber Green fluorescence was tested by plotting fluorescence as a function of temperature to generate a melting curve of the amplicon. The primers used are listed in the Table S1. Each sample was analysed in duplicate using the IQ™-Cycler (Bio-Rad, Hemel Hempstead, UK).

### Migration Assay

Cell migration was performed in Transwell chambers (tissue culture treated, 10 mm diameter, 8 µm pores, Nunc, Rochester, NY or 6.5 mm diameter, 8 µm pores, Costar®, Corning Incorporated, Corning, NY) coated with 100 µg/ml gelatin (0.1% in acetic acid). HUVECs were serum starved overnight in M199 containing 0.5% FBS, pre-treated with the indicated inhibitors for 30 minutes and detached. Cells were resuspended in M199 containing 0.5% bovine serum albumin and the specific inhibitor (or vehicle control) and added to the top of each migration chamber. Cells were then allowed to migrate in the presence of 1 µM S1P or 200 µg/ml HDL_3_ in the lower chamber. Where necessary, each inhibitor was also added in the lower chamber therefore migration was performed in the continuous presence of the inhibitor to be tested. After 4 hours, cells that had not migrated were gently removed by using a cotton swab, whereas migrated cells were fixed with 4% paraformaldehyde, stained with 1% crystal violet and counted using a phase-contrast microscopy. Alternatively, 24 h after transfection with the indicated siRNAs, cells were serum starved overnight in M199 containing 0.5% FBS, then resuspended in M199 containing 0.5% bovine serum albumin and plated on Transwell as above.

### Random Motility Assay

Migration of HUVEC cells was assessed by time-lapse video microscopy. Briefly, 48 hours after transfection, 25×10^3^ cells were seeded in duplicates on 12-well plates. After incubation for 4 h cells were transferred to an inverted phase contrast microscope (Zeiss Welwyn Garden City, UK) with an environmental chamber maintaining a 37°C, 5% CO_2_ atmosphere. Sequential images were collected using a 10× phase contrast objective at intervals of 6 min for a total period of 9 hours. Analysis of motility was determined by measuring the velocity of at least 15 cells/field using the MetaMorph® Software version 7.7 (Downington, PA, USA). Each experiment was performed in duplicate.

### Angiogenesis in vitro

Growth factor-reduced Matrigel™ (BD Biosciences, Oxford, UK) was allowed to polymerise in a 96 well plate for 2 hours at 37°C. Untransfected or transfected HUVECs were serum starved in M199 containing 0.5% FBS overnight. Cells were then detached and plated (10^3^) and 1 µM S1P or 200 µg/ml HDL_3_ were added to the cells where necessary. Alternatively serum starved HUVEC were plated on Matrigel in the presence or absence of 1 µM A66 and the appropriate stimuli. Each experimental condition was performed in triplicate. EC migration and rearrangement was visualised after 4 to 6 hours using an Axiovert200 microscope (10× objective) and the number of branching points counted in five fields covering the whole matrigel surface. Only points generating at least three tubules were counted. Representative fields were photographed using an 10× objective on an Axiovert200 microscope (Zeiss Welwyn Garden City, UK).

### Western Blotting Analysis

Cells were lysed in a buffer containing 1% Triton X-100 supplemented with protease inhibitors and phosphatase inhibitors cocktails (Sigma Adlrich, St.Louis, MO, USA). Protein content was determined using the colorimetric Bradford assay. Proteins were then separated by SDS-PAGE and transferred onto a nitrocellulose membrane. Membrane were saturated at room temperature in PBS containing 5% non fat milk for 1 h at room temperature, washed with PBS containing 0.1% Tween-20, then incubated overnight at 4°C with the primary antibody followed by incubation with peroxidase conjugated anti-mouse or anti rabbit IgG (Sigma Aldrich, St.Louis, MO, USA) for one hour at room temperature. Immunocomplexes were detected by enhanced chemiluminescence (Amersham ECL Plus, GE Healthcare UK Ltd, Little Chalfont, UK). For densitometry analysis, levels of phosphoSer473 Akt and phosphoERK were normalised to levels of actin. Alternatively, membranes were stripped and incubated with total Akt or total ERK and levels of phosphorylated proteins were normalised to total protein levels. Values were then expressed as fold increase over values obtained in cells transfected with scrambled siRNA and unstimulated.

### Caspase-3 Assay

Assay was performed in duplicates following the manufacturer’s instructions (EnzChek® Caspase-3 Assay Kit #2, Molecular Probes®, Life Technologies Ltd, Paisley, UK). Briefly, cells were harvested 48 h after transfection, washed in PBS and then lysed on ice for 30 min. Lysates were centrifuged and transferred to individual micro plates (background fluorescence was determined using lysis buffer). An equal volume of 2× substrate working solution was added to each sample and control, and the plates were incubated in dark at room temperature for approximately 30 minutes. Fluorescence was measured using a microplate fluorometer (FluoStar Optima, BMG LABTECH, GmbH). Fluorescence units were normalised by the total protein content.

### Annexin V Staining

HUVEC were harvested 48 hours after transfection and stained with FITC Annexin V apoptosis detection kit I (BD Biosciences, Oxford, UK) according to manufacturer instructions. Cells positive for Annexin V and negative for propidium iodide were gated. Cells were analysed by flow cytometry using a FACScalibur flow cytometer (BD Biosciences, Oxford, UK) and cellQuest software for data analysis.

### MTT Assay

Assay was performed as previously described [25]. Briefly, 24 h post-transfection HUVEC were incubated in serum free media for further 24 h. Alternatively, HUVEC were incubated for 24 h in serum free with or without 1 µM S1P or in the presence of serum as control. 3-(4,5-Dimethyldiazol-2-yl)-2,5-diphenyl tetrazolium bromide (Sigma Adlrich, St.Louis, MO, USA) solution in M199 (500 µg/ml final concentration) was added to each well for the last 5 h. After washing, DMSO was added to the wells for 15 min, collected and absorbance at 570 nm was determined by using a microplate reader.

### Metabolic Labelling and HPLC Analysis of PtdIns3*P* Levels

HUVEC were labelled with 5 µCi/well of [^3^H]*myo*-inositol (PerkinElmer, Waltham, MA, USA) in inositol free-media M199 containing 0.5% FBS for 24 h. After one wash in PBS, cells were incubated for 15 min in serum free M199 before stimulation with 1 µM S1P for the indicated times. Cells were then lysed with 1 M HCl supplemented with 1 mM TTBSA and phosphoinositides were extracted, deacylated and analysed by HPLC, as previously described [4].

### Statistical Analysis

Data are expressed as means ± SEM. Unless otherwise stated, differences between groups were analysed by Student’s *t* test in Excel (paired, one-tailed distribution) and p<0.05 was considered statistically significant.

## Results

### Different PI3K Isoforms are Involved in Regulation of EC Migration Induced by S1P and HDL_3_

It has been reported that S1P is able to stimulate migration of HUVEC in a mechanism involving different class I PI3K isoforms [22]. The authors however showed that there is also a component of cell migration that is not inhibited by the classical PI3K inhibitor wortmannin. Since class II PI3Ks are more resistant to classical PI3K inhibitors [1], [5], [6], [23] and they have been involved in migration induced by lysophospholipids [1], [5], [6], we decided to investigate whether these PI3K isoforms might also be required for the S1P-induced cell migration.

**Figure 1:**
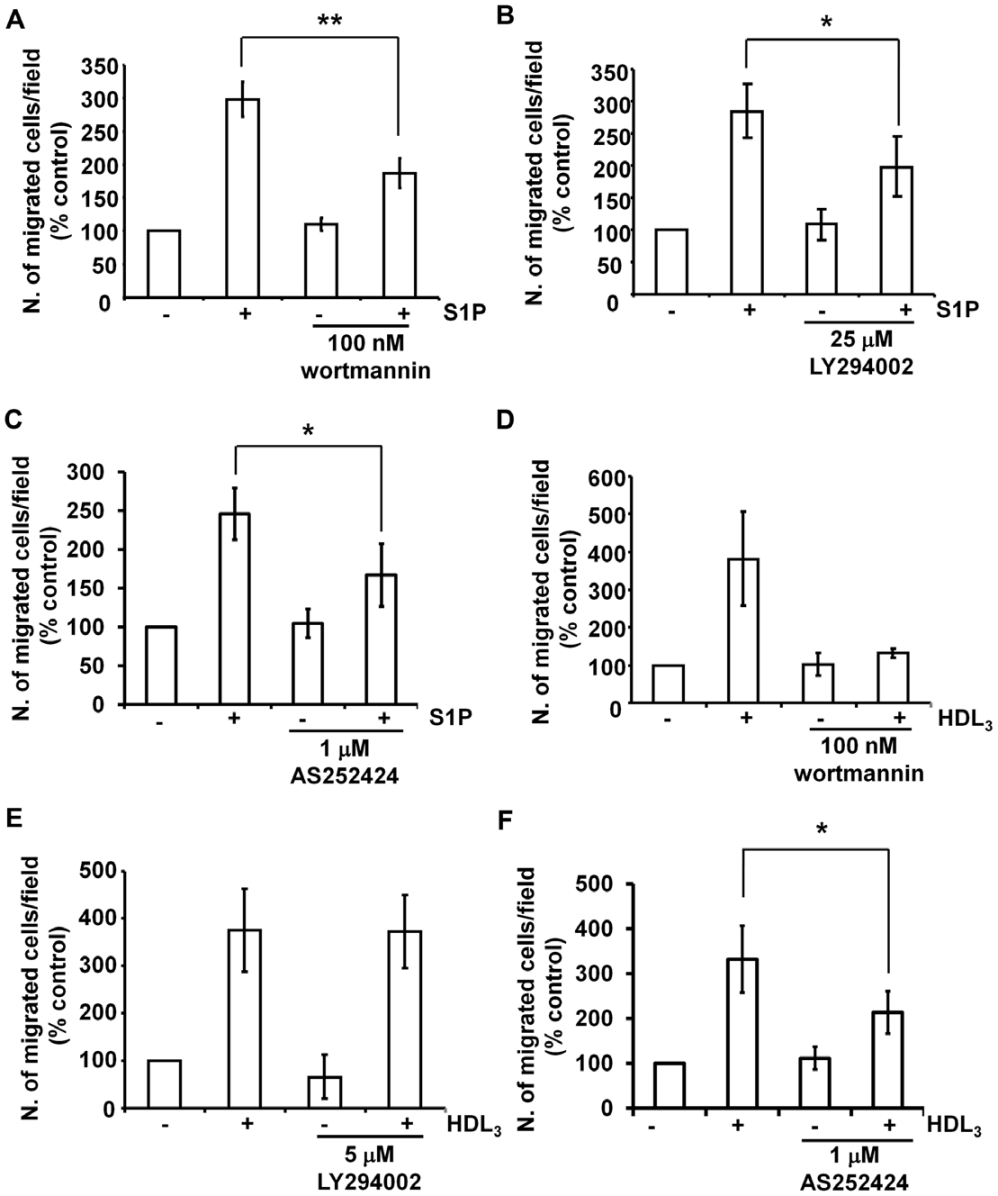
Effect of PI3K inhibitors on S1P- and HDL_3_-induced EC migration. Serum-starved HUVEC were pre-treated with 100 nM wortmannin (A,D), 25 µM LY294002 (B), 5 µM LY294002 (E) or 1 µM AS252424 (C,F) for 30 min. Cell migration induced by S1P (A–C) or HDL_3_ (D-F) was determined by Transwell assays. Briefly, cells resuspended in the presence or absence of the specific inhibitor (or vehicle control) were allowed to migrate in the presence of 1 µM S1P or 200 µg/ml HDL_3_ in the lower chamber for 4 h. Where necessary, each inhibitor was also added in the lower chamber therefore migration was performed in the continuous presence of the inhibitor to be tested. After 4 h, migrated cells were fixed with 4% paraformaldehyde, stained with 1% crystal violet and counted using a phase-contrast microscopy. Data are expressed as percentage of control (cells untreated and unstimulated) and are means ± SEM from 12 (A), 6 (B), 7 (C), 4 (D), 3 (E) and 5 (F) independent experiments. *p<0.05, **p<0.001.

Cells were pre-treated with generic or isoform-specific PI3K inhibitors and migration was determined by Transwell assays in the continuous presence of the inhibitors. Consistent with published data, these experiments revealed that treatment with 100 nM wortmannin only partially inhibited the S1P-induced migration of HUVEC (Figure 1A). Similarly, a partial inhibition of migration was detected upon treatment with 25 µM of the reversible PI3K inhibitor LY294002 (Figure 1B). A detailed analysis of the effect of LY294002 on this process revealed that treatment with up to 5 µM did not affect migration (Figure S1A,B) whereas a slight inhibition was detected upon treatment with 10 µM LY294002 (Figure S1C). None of these treatments affected the basal migration (Figure 1A,B, Figure S1A–C). These results indicated that the S1P-dependent migration was only partially reduced by classical, generic PI3K inhibitors.

It has been previously reported that the class I PI3K isoforms p110β and p110γ are specifically involved in the S1P-induced migration of HUVEC [22]. To assess the role of these isoforms in our experimental conditions HUVEC were first treated with inhibitors that mainly target the class I PI3K isoform p110γ. A partial, but significant inhibition of the S1P-induced migration was detected in cells upon treatment with the compounds AS252424 (Figure 1C) and AS605240 (Figure S1D). Interestingly, in contrast with reported data, in these experimental conditions, no inhibition of cell migration was detected when cells were treated with the specific p110β inhibitor TGX221 (Figure S1E). As for wortmannin and LY294002, none of these treatments affected the basal migration (Figure 1C, Figure S1D,E). The partial inhibitory effect of AS252424 and AS605240 on S1P-induced migration is consistent with the partial inhibition observed using wortmannin and LY294402 and indicates that p110γ is required for S1P-induced cell migration but it is not the only enzyme involved in regulation of this process. This conclusion supports the hypothesis that PI3Ks more resistant to generic inhibitors may be also involved in this process.

Since S1P is a key component of HDL, we then investigated the effect of PI3K inhibitors on HDL_3_-induced cell migration. Consistent with data obtained with S1P alone, no increase in migration was detected in cells upon treatment with wortmannin (Figure 1D) whereas 5 µM LY294002 did not affect this process (Figure 1E) and treatment with AS252424 partially blocked the HDL_3_-induced migration (Figure 1F).

Taken together these data indicate that S1P and HDL_3_ induce HUVEC migration in a mechanism which is either partially independent on PI3K or involves activation of PI3Ks more resistant to treatment with the classical inhibitors.

### Class II PI3K Isoforms are Involved in S1P- and HDL-induced Cell Migration

**Figure 2:**
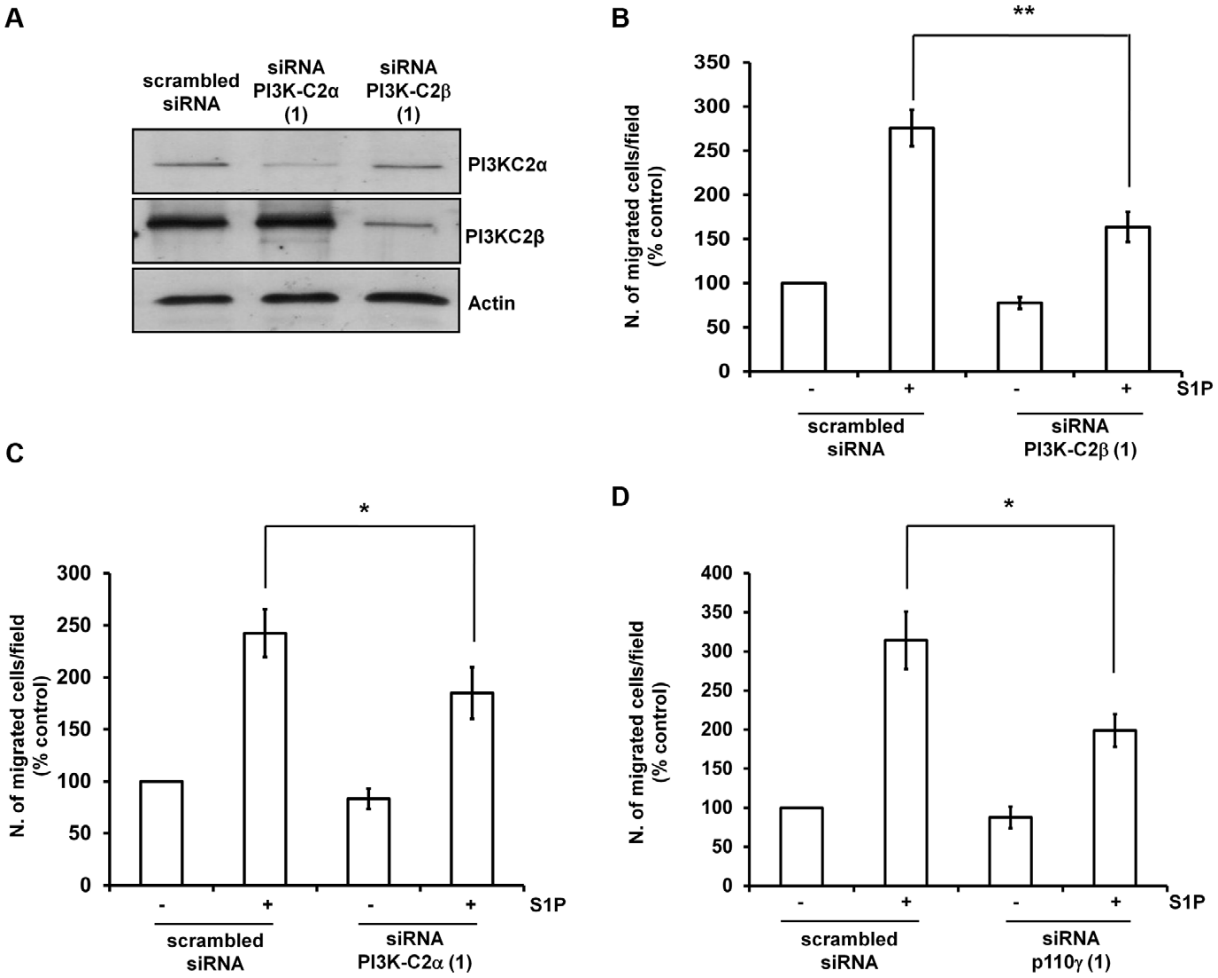
Class II PI3Ks are involved in S1P-dependent EC migration. (A) Expression levels of PI3K-C2α and PI3K-C2β in HUVEC transfected with a scrambled siRNA or siRNAs (“Sequences 1”) specifically targeting the indicated enzymes. (B-D) Twenty four hours after transfection with the indicated siRNAs cells were serum starved overnight and S1P-mediated cell migration was determined by Transwell assays as above. Data are expressed as percentage of migration of cells transfected with scrambled siRNA and unstimulated (control) and are means ± SEM from 17 (B), 11 (C) and 6 (D) independent experiments. *p<0.05; **p<0.001.

As mentioned above, one of the features of class II isoforms PI3K-C2α and PI3K-C2β compared to class I enzymes is their peculiar resistance to treatment with classical PI3K inhibitors [1], [6]. In order to determine whether class II PI3Ks have a role in regulation of S1P- and HDL_3_-dependent migration in HUVEC, we decided to investigate the effect of downregulation of their protein expression on these processes using specific siRNAs. First we checked whether transfection of HUVEC affected the S1P-induced cell migration by using a non-targeting, “scrambled” siRNA as control. Transfection of HUVEC reduced the number of migrated cells compared to untransfected cells both in unstimulated and in stimulated conditions (Figure S2A), as expected from cells treated with transfection reagent. Nevertheless, transfected cells were still able to respond to S1P stimulation in a manner which was comparable to untransfected cells (Figure S2A) indicating that the transfection procedure does not affect the ability of HUVEC to migrate upon S1P stimulation. We then investigated the S1P-dependent migration in HUVEC upon PI3K-C2α and PI3K-C2β downregulation. Two distinct siRNAs were used to downregulate the expression levels of each class II PI3K isoform (Figure 2A, Figure S2B). Data revealed that downregulation of PI3K-C2β specifically reduced the S1P-mediated cell migration (Figure 2B and Figure S2C). A reduction in cell migration upon S1P stimulation was also detected in cells upon downregulation of PI3K-C2α (Figure 2C and Figure S2D). Interestingly, we observed a slight but significant inhibition of S1P-induced migration in cells treated with 1 µM of the compound A66 (Figure S2E). It has been reported that A66 is a specific inhibitor of the class IA PI3K isoform p110α (IC_50_ 32 nM), able to reduce Akt phosphorylation in specific cancer cells at concentrations in the nanomolar range [26]. The observation that PI3K-C2β is the only other PI3K isoform to be inhibited by this compound with an IC_50_ in the nanomolar range (462 nM) [26] and that treatment of HUVEC with 1 µM A66 does not inhibit the S1P-induced Akt phosphorylation (described below) may suggest that the detected effect on cell migration is due to at least a partial inhibition of PI3K-C2β.

Consistent with previously reported data [22] and with our experiments using AS252424 and AS605240 (Figure 1C and Figure S1D), downregulation of p110γ using two distinct siRNAs also inhibited the S1P-dependent cell migration (Figure 2D, Figure S2F). Efficiency of p110γ knock down was assessed by qPCR analysis (Figure S3A,B). This analysis also confirmed that downregulation of each PI3K did not affect the mRNA levels of the others (Figure S3B-D). Taken together these data reveal that class II PI3Ks and p110γ are involved in EC migration induced by S1P.

**Figure 3:**
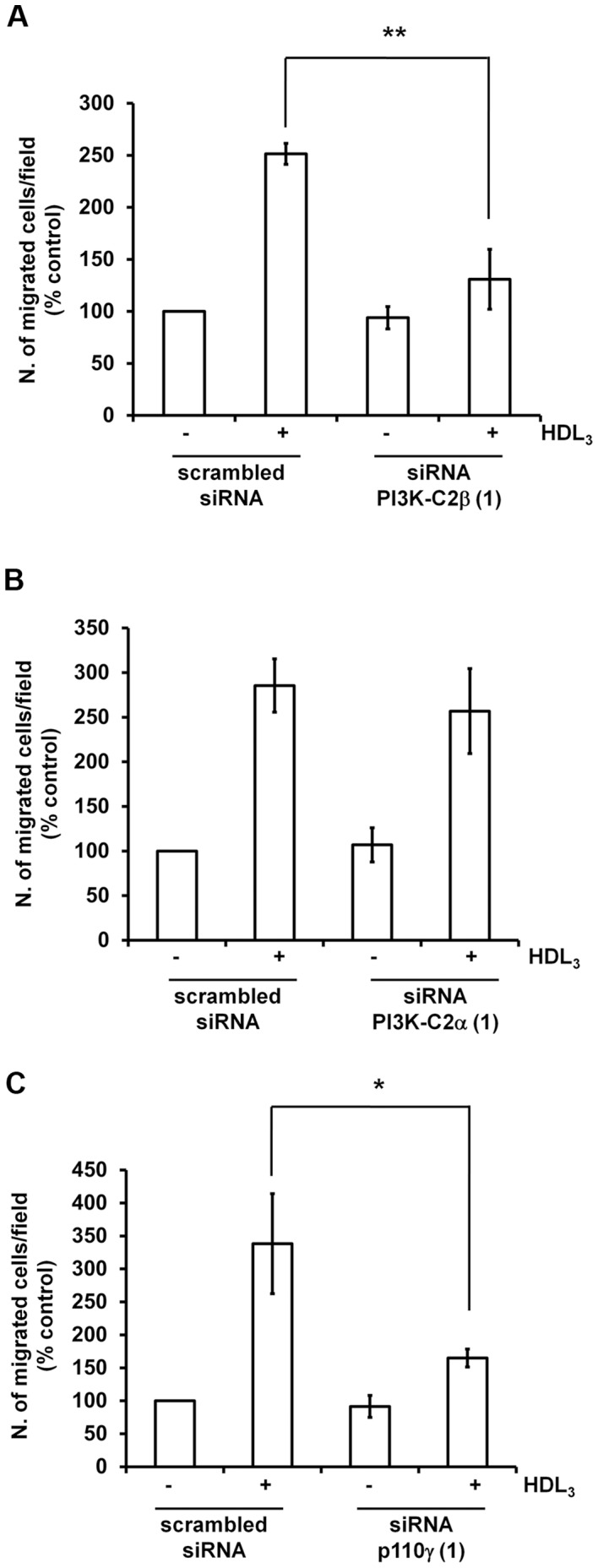
Class II PI3Ks are involved in HDL_3_-dependent EC migration. Twenty four hours after transfection with the indicated siRNAs cells were serum starved overnight and HDL_3_-mediated cell migration was determined by Transwell assays as above. Data are expressed as percentage of migration of cells transfected with scrambled siRNA and unstimulated (control) and are means ± SEM from 4 (A), 5 (B) and 5 (C) independent experiments. *p<0.05; **p<0.01.

We then investigated whether these PI3K isoforms had also a role in HDL_3_-induced cell migration. Data demonstrated that knockdown of PI3K-C2β indeed inhibited cell migration upon HDL_3_ stimulation (Figure 3A) whereas downregulation of PI3K-C2α did not appear to affect this process (Figure 3B). Consistent with data obtained with the inhibitors, knock down of p110γ also resulted in significant inhibition of HDL_3_-dependent migration (Figure 3C).

Taken together these data indicate that class II PI3Ks (mainly PI3K-C2β) and p110γ regulate EC migration induced by both S1P and HDL_3_.

### Class II PI3K Isoforms Regulate EC Morphogenesis

**Figure 4:**
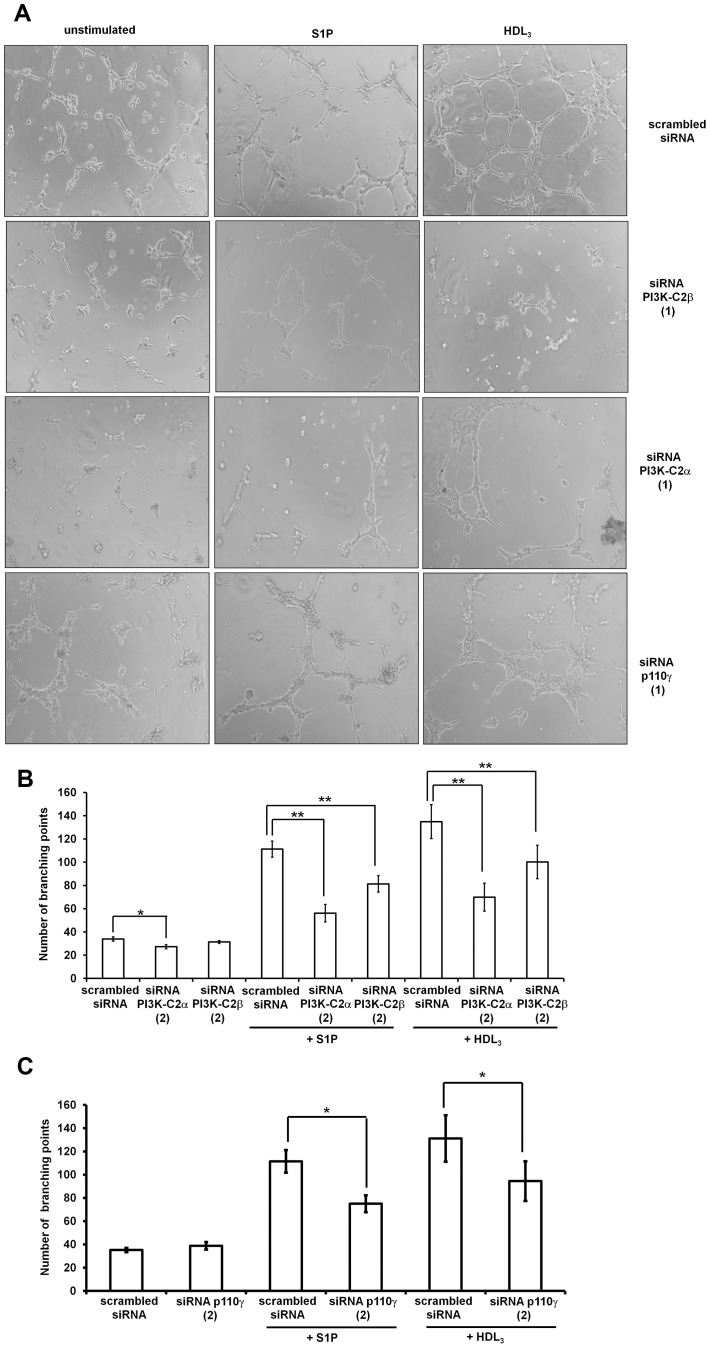
Class II and class IB PI3Ks regulate remodelling of HUVEC. HUVEC transfected with the indicated siRNAs were serum starved in M199 containing 0.5% FBS overnight before being detached and plated on growth factor reduced Matrigel in the presence of 1 µM S1P or 200 µg/ml HDL_3_. EC rearrangement was visualised after 4 to 6 h using an Axiovert200 microscope. (A) Representative images of branching points formation in HUVEC transfected with the indicated siRNAs (Sequences 1) in the absence or presence of S1P or HDL_3_. (B,C) Results from quantitative analysis in HUVEC transfected with the indicated siRNAs (Sequences 2) in the absence or presence of S1P or HDL_3_. Data indicate the total number of branching points and are means ± SEM from 4 (B) and 3 (C) independent experiments. *p<0.01; **p<0.001.

Since migration is a crucial process in EC shape change and morphogenic rearrangement on Matrigel we then analysed the effect of downregulation of the PI3K isoforms on this process. Images of unstimulated, S1P- and HDL_3_-stimulated HUVEC from one representative experiment are shown in Figure 4A. Downregulation of either PI3K-C2α or PI3K-C2β using two distinct siRNAs reduced HUVEC morphogenesis upon stimulation with S1P and HDL_3_ (Figure 4B, Figure S4A). Inhibition on the basal remodelling of the cells (induced by interaction with Matrigel and likely by factors released by the cells) was also consistently detected in cells upon downregulation of PI3K-C2α (Figure 4B, Figure S4A). Parallel experiments also indicated a reduction in the number of branching points in cells upon downregulation of p110γ using two distinct siRNAs following stimulation with either S1P or HDL_3_ but not under basal conditions (Figure 4C, Figure S4B,C). In addition, consistent with data obtained in migration experiments, treatment of HUVEC with 1 µM A66 significantly inhibited both the HDL_3_- and S1P-induced remodelling on Matrigel (Figure S4D,E).

Taken together these data indicate that a concerted action of distinct PI3K isoforms is required to regulate the complex process of EC morphogenesis on extracellular matrix.

### Different PI3K Isoforms Control Distinct, not Redundant Processes Regulating EC pro-Angiogenic Phenotype

**Figure 5:**
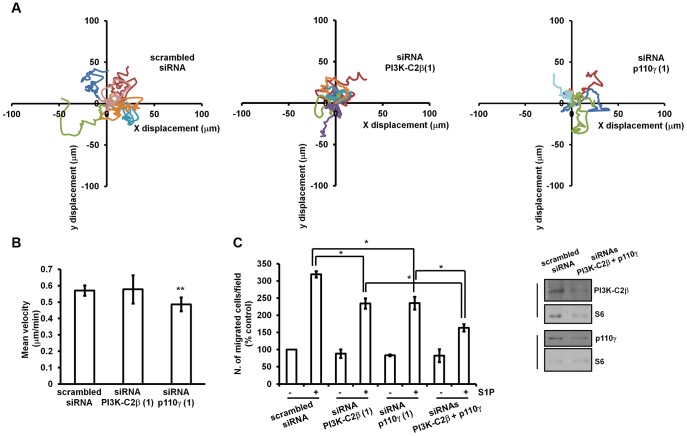
PI3K-C2β and p110γ play distinct role in regulation of S1P-dependent cell migration. Random motility of HUVEC transfected with the indicated siRNAs was monitored as described in the Materials and Methods section. (A) Representative track paths throughout time of 6 cells are shown (8 h). (B) Data indicate the mean velocity/min and are means ± SEM from 3 independent experiments. **p<0.01 vs cells transfected with scrambled siRNA. (C) Results from Transwell assays performed in HUVEC transfected with the indicated siRNAs. Data are expressed as percentage of migration of cells transfected with scrambled siRNA and unstimulated (control) and are means ± SEM from 3 independent experiments. *p<0.01. Downregulation of PI3K-C2β and p110γ was confirmed by Western blotting.

Our data so far indicate that three different PI3K isoforms participate in EC morphogenesis. Remodelling of EC on Matrigel is the result of several cellular functions, including migration, proliferation and survival. We therefore decided to investigate in more details the specific contribution of each enzyme to this process. Our data have shown that p110γ and PI3K-C2β are mainly involved in both S1P- and HDL_3_-induced cell migration. To gain further insight into the specific role of each isofom in regulation of cell migration we performed random motility assays. Specifically, we measured the mean velocity of cells in serum by tracking individual HUVEC transfected with siRNAs targeting each distinct PI3K or with the control scrambled siRNA. Representative track paths are shown in Figure 5A. A significant reduction of the mean velocity was detected in cells upon p110γ but not PI3K-C2β downregulation (Figure 5B), suggesting that the two PI3Ks may control different steps of cell migration.

To further investigate the hypothesis that p110γ and PI3K-C2β contribute to cell migration by regulating distinct functions, we decided to analyse the effect of combined downregulation of p110γ and PI3K-C2β on S1P-induced cell migration. Consistent with data previously presented, knockdown of either PI3K-C2β or p110γ alone significantly inhibited the S1P-induced migration (Figure 5C). Importantly migration was further inhibited when PI3K-C2β and p110γ were downregulated simultaneously, indicating that blockade of PI3K-C2β and p110γ together has a more pronounced inhibitory effect on S1P-dependent cell migration. These data support the hypothesis that p110γ and PI3K-C2β have distinct, non redundant roles in regulation of cell migration.

**Figure 6:**
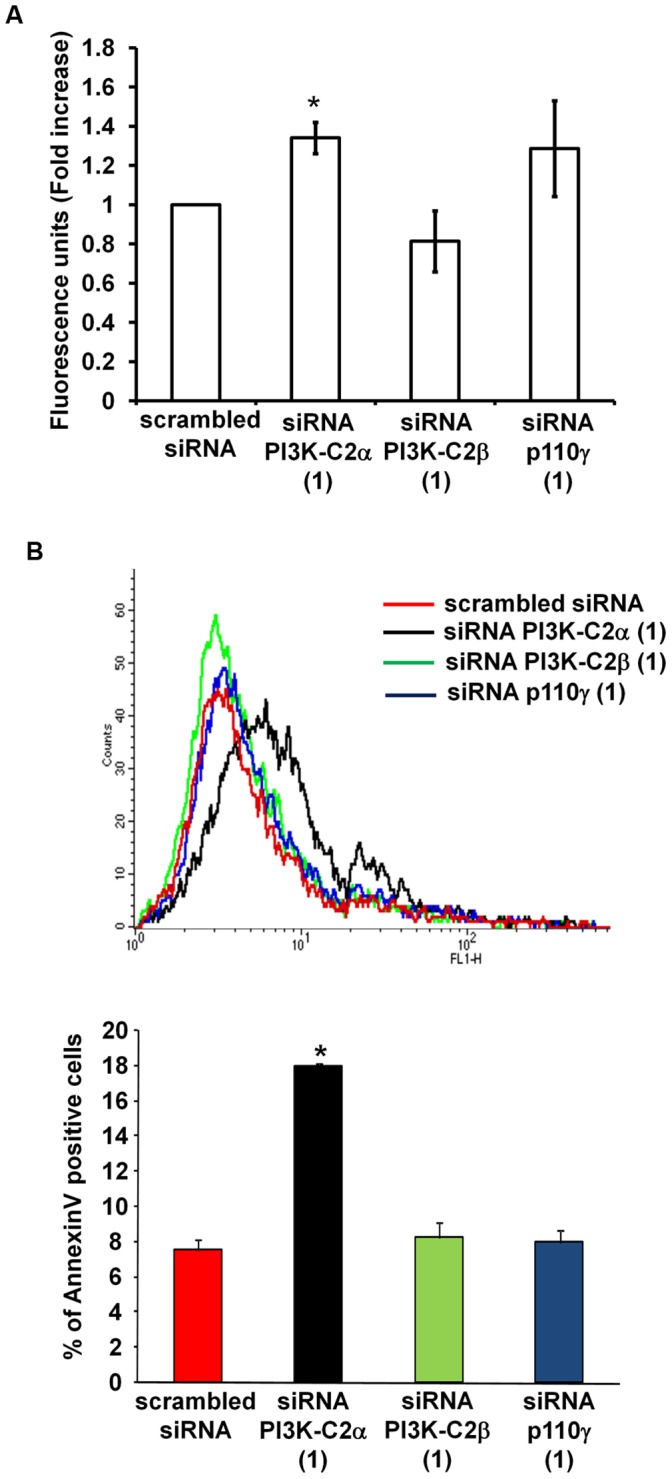
Effect of PI3Ks downregulation on EC apoptosis. (A) Results from caspase 3 assay performed on lysates from HUVEC obtained 48 h after transfection with the indicated siRNAs. Data are means ± SEM from 3–4 independent experiments. Student’s t-Test: un-paired *p<0.05 vs cells transfected with PI3K-C2β siRNA. (B) HUVEC were transfected with the indicated siRNAs. After 48 h the percentage of apoptotic cells was determined by Annexin V staining. Annexin V positive and propidium iodide negative cells were gated. Results are expressed as means ± SEM from 3 independent experiments. *p<0.05 vs scrambled siRNA treated cells.

Our data so far indicated that PI3K-C2α is also critical for EC morphogenesis. We therefore sought to determine the specific role of PI3K-C2α in this process. Since downregulation of PI3K-C2α had a smaller effect compared to PI3K-C2β knockdown (in the case of S1P) or no effect at all (in the case of HDL_3_) on migration assessed by Transwell assays, we investigated whether downregulation of PI3K-C2α affected other cellular function(s) required for EC remodelling. It has been recently reported that PI3K-C2α knockdown reduces viability [27] and augmented apoptosis of HUVEC [7]. Consistent with this, we observed that downregulation of PI3K-C2α using two distinct siRNAs induced apoptosis in HUVEC in the presence of serum as assessed by caspase 3 assay (Figure 6A, Figure S5A) and by Annexin V staining (Figure 6B) whereas no effect was observed upon silencing of PI3K-C2β (Figure 6, Figure S5A). Consistent with these data, downregulation of PI3K-C2α but not PI3K-C2β reduced the viability of HUVEC upon serum starvation (Figure S5B). Although results from caspase assay seemed to suggest a small increase in apoptosis upon downregulation of p110γ, values did not reach statistical significance (Figure 6A). Moreover no increase in the number of Annexin V-positive cells was detected in cells lacking p110γ (Figure 6B). Importantly, these data indicate that downregulation of PI3K-C2α induces apoptosis of HUVEC even in the presence of serum (Figure 6). These data suggest that PI3K-C2α but not PI3K-C2β or p110γ primarily regulates HUVEC survival, further supporting the hypothesis that the distinct PI3Ks can regulate EC morphogenesis by controlling different processes.

### Downregulation of Class II PI3Ks does not Prevent the S1P- or HDL_3_-dependent Phosphorylation of Akt or ERK

**Figure 7:**
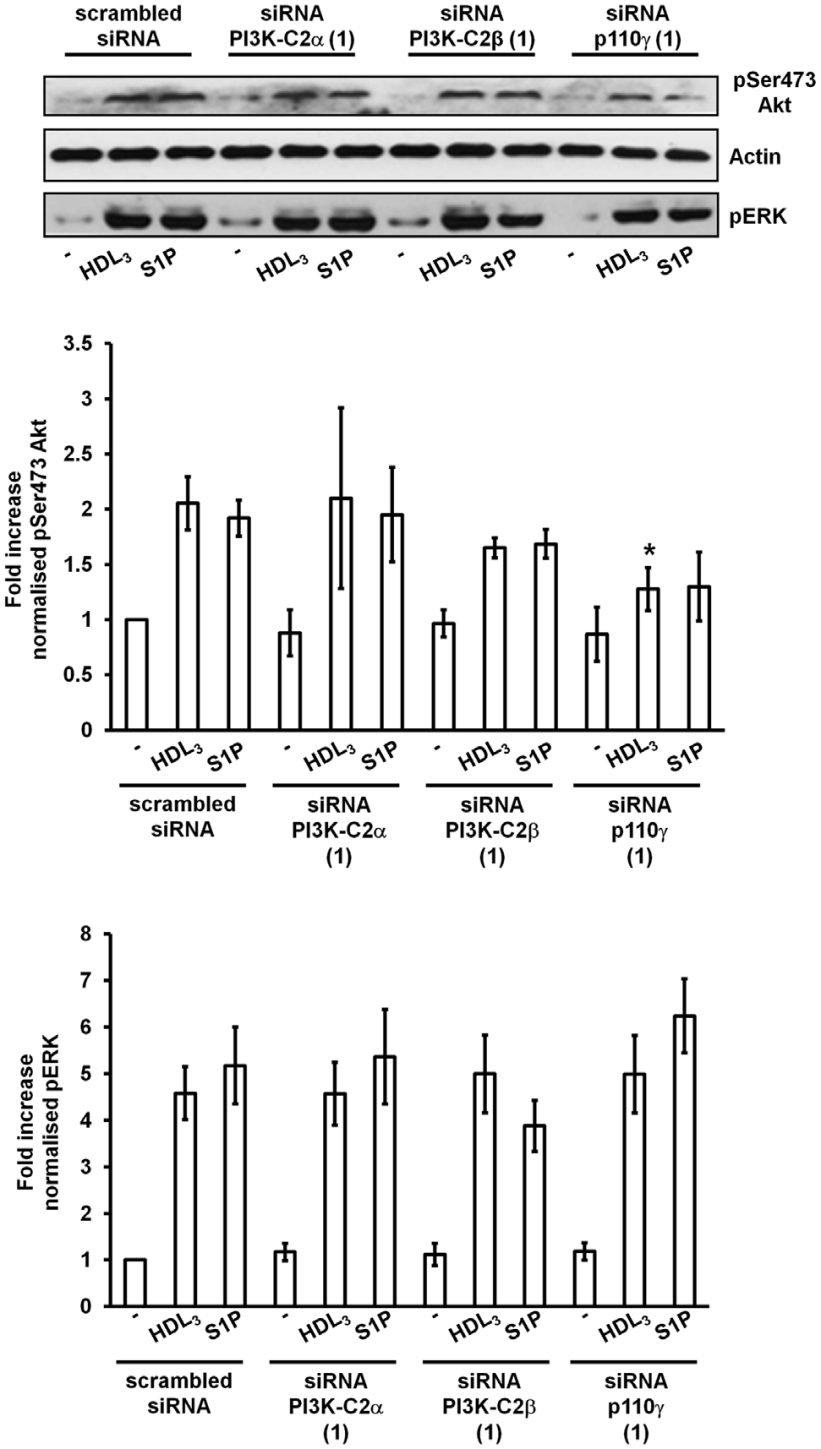
Class II PI3Ks do not regulate Akt or ERK activation. HUVEC transfected with the indicated siRNAs were serum starved in M199 containing 0.5% FBS overnight before stimulation with HDL_3_ or S1P for 10 min. Akt and ERK phosphorylation was assessed by Western blotting. Densitometry analysis shows means ± SEM from 3–4 (Akt) and 4 (ERK) independent experiments.

It has been reported that Akt activation is important for EC migration [21], [25] and capillary tube formation [25]. Furthermore it has been shown that inhibition of ERK activation is able to partially inhibit S1P-induced EC migration [22]. We therefore investigated whether class II PI3Ks have a role in S1P- or HDL_3_-dependent activation of ERK and Akt. Downregulation of PI3K-C2α with two distinct siRNAs did not affect the S1P-dependent ERK and Akt phosphorylation at its residue Ser473 (Figure 7, Figure S6A). Phosphorylation of both enzymes upon S1P stimulation was also clearly detectable in cells lacking PI3K-C2β (Figure 7, Figure S6B). Consistent with this, treatment of HUVEC with 1 µM A66, a concentration previously shown to be able to inhibit the S1P-induced cell migration (Figure S2E), did not inhibit the S1P-induced Akt or ERK phosphorylation (Figure S6C). Knockdown of either PI3K-C2α or PI3K-C2β did not affect the HDL_3_-mediated ERK or Akt phosphorylation (Figure 7). No inhibition of ERK phosphorylation was observed in cells transfected with siRNA targeting p110γ (Figure 7) as previously reported [22] whereas downregulation of p110γ slightly affected the HDL_3_-dependent and possibly the S1P-dependent Akt phosphorylation, although values from densitometry analysis did not reach significance in this latter case (Figure 7). On the other hand treatment with classical PI3K inhibitors wortmannin and LY294002 and the p110β inhibitor TGX221 completely blocked the S1P-induced Akt but not ERK phosphorylation (Figure S6D), consistent with previous report [22]. Interestingly, in these experimental conditions treatment with either AS252424 or AS605240 was also able to inhibit Akt but not ERK phosphorylation upon S1P stmulation (Figure S6E).

These data indicate that class II PI3Ks do not regulate the S1P- and HDL_3_-dependent Akt and ERK activation further supporting the conclusion that class I and class II PI3Ks can activate distinct signalling pathways. Interestingly, HPLC analysis of phosphoinositides extracted from [^3^H]*myo*-inositol-labelled HUVEC revealed a very rapid and transient increase in the levels of PtdIns3*P*, the main lipid product of class II PI3Ks, upon S1P stimulation (Figure S7). Future studies will focus on the identification of the specific downstream effectors of PtdIns3*P*.

## Discussion

In this study we investigated the relative contribution of different PI3K isoforms to EC functions specifically regulated by the sphingolipid S1P and the lipoprotein HDL_3_. Previous work had demonstrated that EC migration upon stimulation with S1P requires both class I isoforms p110β and p110γ [22]. While the contribution of these two isoforms is necessary for this process to occur, data also suggested the existence of other signalling pathways involved in this process. Based on evidence provided in the previous study [22] and confirmed by our own results showing that the S1P-dependent EC migration is only partially inhibited by treatment with wortmannin and LY294002 we hypothesised that class II PI3Ks could also be involved in this process. Indeed class II PI3Ks are more resistant to treatment with these classical PI3K inhibitors compared to class I PI3Ks [1], [6]. Furthermore we previously reported that the class II enzyme PI3K-C2β can be activated by the lysophospholipid LPA [5].

Our results demonstrated that downregulation of class II PI3Ks inhibits the S1P-induced EC migration. By using two distinct siRNAs and two inhibitors that mainly target p110γ we also confirmed the involvement of p110γ in this process, consistent with previous report [22]. We further showed that PI3K-C2β and p110γ play also a role in migration induced by treatment with HDL_3_. More important, our data clearly demonstrated that all three PI3K isoforms PI3K-C2α, PI3K-C2β and p110γ contribute to EC morphogenesis since downregulation of each of these enzymes strongly prevents EC shape change and remodelling on Matrigel.

These data indicated that a concerted action of distinct PI3Ks is necessary to tightly regulate EC morphogenesis and raised the question of whether these isoforms play redundant roles or they regulate distinct processes ultimately controlling EC remodelling. A more detailed analysis revealed that downregulation of PI3K-C2β and p110γ specifically affects morphogenesis induced by treatment with either S1P or HDL_3_ whereas downregulation of PI3K-C2α appears to inhibit the basal as well as the stimulated cell remodelling. Inhibition of basal as well as vascular endothelial growth factor (VEGF)-A- and serum-induced remodelling was recently reported in HUVEC upon PI3K-C2α but not PI3K-C2β downregulation [7]. These results may suggest distinct mechanisms of activation of the two class II isoforms with a potential selective activation of PI3K-C2β downstream of GPCR activation. This would be consistent with our previous data demonstrating that PI3K-C2β but not PI3K-C2α is involved in LPA-dependent migration of cancer cells [5] but further studies are required to validate this hypothesis.

We then investigated the specific contribution of each PI3K isoform to EC shape change and morphogenesis. The switch of EC towards a pro-angiogenic phenotype is the result of several intracellular events such as cell proliferation, survival and migration. Our results support the hypothesis that the contribution of PI3K-C2β and p110γ to EC remodelling is mostly associated to their role in regulation of cell migration induced by either S1P or HDL_3_. Although both enzymes control HUVEC migration, their specific roles seem not to be overlapping. Indeed inhibition of p110γ but not PI3K-C2β was able to slightly but significantly affect cell speed assessed by random motility assays, suggesting that PI3K-C2β and p110γ may regulate EC migration by controlling distinct steps in this process. Moreover we observed that simultaneous downregulation of p110γ and PI3K-C2β inhibits the S1P-induced migration to a greater extent than downregulation of each PI3K isoform alone further supporting the hypothesis that the two enzymes have non redundant roles in regulation of cell migration.

The contribution of PI3K-C2α to EC remodelling appears to be mainly associated to its role in regulation of EC survival. Our data showed that downregulation of this enzyme induces apoptosis in HUVEC even in the presence of serum, suggesting that the reduced morphogenetic response may be attributed to intrinsic increased apoptosis in cells lacking PI3K-C2α. Interestingly we observed that treatment of HUVEC with 1 µM S1P was not able to induce survival of serum starved HUVEC (Figure S5C). This observation, together with data showing that knockdown of PI3K-C2α consistently reduced the basal as well as the stimulated EC remodelling further supports the hypothesis that the detected inhibition of EC remodelling is at least partially due to the intrinsic reduced viability of the cells upon downregulation of PI3K-C2α. Several studies have reported a role for PI3K-C2α in regulation of cell viability and/or survival [1], [9]. Relevant to our own study it was previously reported that PI3K-C2α is critical for HUVEC viability [27]. Furthermore it has been recently shown that downregulation of this enzyme increases apoptosis of HUVEC upon serum and growth factors deprivation or upon treatment with staurosporine [7]. More important retinas from tamoxifen-inducible EC-specific PI3K-C2α depleted mice showed a 5.5-fold increase in apoptosis compared to control littermates [7].

Taken together results from this study support the hypothesis that a concerted action of distinct PI3K isoforms is required to regulate the complex process of EC remodelling. The precise mechanisms by which the enzymes are able to control this process are still not completely defined. It has been previously proposed that p110γ can control EC migration through regulation of Rac1 activation [22]. Interestingly Rac1 has also been identified as a downstream effector of PI3K-C2β in regulation of cell migration [28]. On the other hand the specific effect of PI3K-C2α downregulation on the VEGF-A-dependent EC functions has been at least partially ascribed to a defect in RhoA activation [7]. It remains to be addressed whether Rac1 or RhoA are also involved in the S1P- and HDL_3_-mediated EC remodelling.

One of the main mechanisms by which class I and class II PI3Ks can activate distinct signalling pathways is through generation of different lipid products. Indeed, while it is well established that class I PI3Ks mainly catalyse the synthesis of PtdIns(3,4,5)*P_3_* increasing evidence supports the conclusion that PtdIns3*P* is the main lipid product of class II PI3Ks [1], [3], [4], [7], [9]. Interestingly we demonstrated that LPA specifically increases the levels of PtdIns3*P* in ovarian and cervical cancer cells in a mechanism dependent on activation of PI3K-C2β [5]. A time course analysis revealed a very rapid and transient increase in PtdIns3*P* levels upon S1P stimulation of HUVEC (Figure S7). Being a distinct phosphoinositide compared to the main product of class I PI3Ks PtdIns(3,4,5)*P_3_*, it is very likely that PtdIns3*P* activates distinct downstream effectors and therefore distinct cellular pathways. In this respect it is noteworthy that we observed that downregulation of class II PI3Ks did not affect the S1P- or the HDL_3_-dependent activation of Akt, one of the main downstream effectors of class I PI3Ks/PtdIns(3,4,5)*P_3_* pathway. The question of whether class II PI3Ks are able to activate Akt has been critical to address the issue of potential redundancy between these enzymes and class I PI3K isoforms [1], [9]. Our data here firmly demonstrate that neither PI3K-C2α nor PI3K-C2β mediates Akt activation upon S1P and HDL_3_ stimulation. On the other hand treatment of HUVEC with classical PI3K inhibitors or the p110β specific inhibitor TGX221 was able to completely block the S1P-induced Akt phosphorylation, consistent with previous data [22]. Interestingly it has also been demonstrated that PI3K-C2α does not regulate the VEGF-A-dependent Akt activation [7].

Taken together these data strongly support the conclusion that class I and class II PI3Ks activate distinct intracellular pathways and therefore they play non redundant intracellular roles. Studies using animal models are also now providing evidence supporting this conclusion. It is well established that class I PI3Ks play a key role in EC functions [29]. Knock out mice for the class IA catalytic subunit p110α die early during embryogenesis (E9.5) and knock-in strategies highlighted a critical role for this isoform in vascular development [30]. A very recent and elegant study has now revealed that PI3K-C2α has also a pivotal role in angiogenesis and vascular development [7]. Homozygous global PI3K-C2α-deleted embryos die between E10.5-E11.5 because of defects in vascular formation [7]. Endothelial specific ablation of PI3K-C2α also resulted in embryonic lethality highlighting a critical and specific role for endothelial PI3K-C2α in normal vascular formation and development. This study demonstrated that PI3K-C2α has a pivotal role in regulation of angiogenesis and vascular barrier integrity and highlighted a non redundant role of this isofom in mouse development [7]. Studies also revealed that capillarisation and arteriogenesis following unilateral limb ischemia were reduced in muscles from PI3Kγ^−/−^ compared to wild type mice, resulting in delayed blood flow recovery in knock out animals [31] further supporting the evidence for involvement of different PI3Ks in EC functions. As for PI3K-C2β a study reporting a knock out model for this enzyme specifically focussed on investigation of a potential role for PI3K-C2β in epidermal differentiation and no further investigation of the potential effects on ECs was performed [32].

Finally, the physiological relevance of our study is reinforced by analysis of EC functions induced by high density lipoproteins, the most relevant carrier of S1P in plasma. Our data demonstrate that class II PI3Ks are also involved in EC angiogenic response induced by HDL_3_. Numerous epidemiological studies have shown an inverse correlation between plasma HDL cholesterol level and the risk of coronary artery disease [33], [34]. The beneficial effect of these particles is related to their ability to remove cholesterol from peripheral tissues and to protect against the development of endothelial dysfunction regulating a wide range of cellular activities [35]. For instance HDL has been shown to promote prostacyclin and nitric oxide synthesis [36], [37], to inhibit leukocytes adhesion and platelets aggregation and to stimulate EC proliferation and migration, crucial to both neovascularisation and to a successful response to vascular injury [38], [39]. The precise mechanisms and the HDL components underlying these atheroprotective effects are still under investigation but apolipoprotein A-I, the predominant HDL apolipoprotein, and several lysosphingolipids such as S1P, sphingosylphosphorylcholine and lysosulfatide have been identified as possible candidates. In particular S1P is specifically concentrated in the small dense HDL_3_ particles [14] and several studies have shown that HDL-induced EC migration and morphogenesis involves both the scavenger receptor class B member 1 (SR-B1) and S1P receptors EDG-1/S1P_1_ and EDG-3/S1P_3_ [39]–[41], suggesting that HDL binds SR-B1 on the cell surface bringing lysophospholipids into direct proximity to their EDG receptors and that S1P may account for the biological activity of this class of lipoproteins [42]. Consistent with this, our data reveal that the ability of HDL_3_ to induce HUVEC migration and branching points formation on Matrigel involves a similar dependence from the three PI3Ks, as for S1P alone.

In conclusion our study has provided novel evidence of a direct involvement of class II PI3Ks in EC functions regulated by S1P and HDL_3_ and it has further indicated that a concerted action of PI3K isoforms playing distinct, non redundant roles is required to modulate EC migration and morphogenesis.

## Supporting Information

Figure S1 **Effect of PI3K inhibitors on S1P- and HDL_3_-induced EC migration.** Serum-starved HUVEC were pre-treated with 1 µM LY294002 (A), 5 µM LY294002 (B), 10 µM LY294002 (C), 1 µM AS605240 (D) or 100 nM of the specific p110β inhibitor TGX221 (D) for 30 min. Cell migration induced by S1P was determined by Transwell assays. Data are expressed as percentage of control (cells untreated and unstimulated) and are means ± SEM from 6 (A), 5 (B), 6 (C), and 3 (D) independent experiments. *p<0.05, **p<0.001.
(TIF)

Figure S2 **Class II and class IB PI3Ks are involved in S1P-induced EC migration.** (A) Results from Transwell assays performed in control, untransfected HUVEC and HUVEC transfected with a scrambled siRNA. Data are expressed as percentage of control (cells untransfected and unstimulated) and are means ± SEM from 6 independent experiments. (B) Levels of PI3K-C2α and PI3K-C2β in cells transfected with specific siRNAs (sequences 2) were assessed by Western blotting. (C-D) Results from Transwell assays performed in HUVEC transfected with the indicated siRNAs. Data are expressed as percentage of each control (cells transfected with each siRNA and unstimulated) and are means ± SEM from 5 (C) and 4 (D) independent experiments.*p<0.05. (E) Results from Transwell assays performed in HUVEC treated with 1 µM of the inhibitor A66. Data are expressed as percentage of control (cells untreated and unstimulated) and are means ± SEM from 4 independent experiments.*p<0.05. (F) Results from Transwell assays performed in HUVEC transfected with a scrambled siRNA or siRNA specifically targeting p110γ (sequence 2). Data are expressed as percentage of each control (cells transfected with each siRNA and unstimulated) and are means ± SEM from 2 independent experiments.
(TIF)

Figure S3 **RT-qPCR analysis of PI3Ks levels.** (A) Downregulation of p110γ mRNA levels using two distinct siRNAs was determined by RT-qPCR. (B) HUVEC were transfected with siRNAs targeting the indicated PI3Ks. Efficiency and specificity of downregulation was determined by RT-qPCR.
(TIF)

Figure S4 **Class II and class IB PI3Ks are involved in remodelling of HUVEC.** (A-C) Results from analysis of EC rearrangement. Data indicate the total number of branching points and are means ± SEM from 3–4 (A), 3 (B), and 4 (C) independent experiments. (D,E) The S1P- and HDL_3_-dependent HUVEC was assessed in the absence or presence of 1 µM A66. Representative images (D) and data indicating the total number of branching points (E) are shown. Data are means ± SEM from 4 independent experiments. **p<0.01.
(TIF)

Figure S5 **Effect of PI3Ks downregulation on EC apoptosis.** (A) Results from caspase 3 assay performed on lysates from HUVEC obtained 48 h after transfection with the indicated siRNAs (sequences 2). Data are means ± SEM from 2 independent experiments. (B) HUVEC transfected with the indicated siRNAs were incubated in serum free M119 after 24 h from transfection. Viability of cells was assessed by MTT assay after further 24 h. Data are expressed as fold increase over control (cells transfected with scrambled siRNA) and are means ± SEM from 4–6 independent experiments. (C) Results from MTT assays performed in HUVEC incubated in serum free M199 or M199 supplemented with 1 µM S1P or 10% FBS. Data are expressed as fold increase over control (cells in serum free media) and are means ± SEM from 5 independent experiments.
(TIF)

Figure S6 **Effect of PI3Ks downregulation and PI3K inhibitors on the S1P-dependent Akt and ERK phosphorylation.** Representative images of Western blotting analysis of Akt and ERK phosphorylation performed in HUVEC. Membranes were then stripped and incubated with the corresponding total antibodies. (A-B) HUVEC transfected with the indicated siRNAs were serum starved overnight before stimulation with 1 µM S1P for 10 min. (C) Serum-starved HUVEC were treated with the indicated concentration of A66 for 30 min before stimulation with 1 µM S1P for 10 min in the presence of the inhibitor. (D) Serum-starved HUVEC were treated with 100 nM wortmannin, 10 µM LY294002 or 100 nM TGX221 for 30 min before stimulation with 1 µM S1P for 10 min in the presence of the inhibitors. (E) Serum-starved HUVEC were treated with 1 µM AS252424 or AS605240 for 30 min before stimulation with 1 µM S1P for 10 min in the presence of the inhibitor.
(TIF)

Figure S7 **S1P induces** ***de novo*** **synthesis of PtdIns3** ***P*** **.** HUVEC were labelled with [^3^H]*myo*-inositol before stimulation with 1 µM S1P for the indicated times. Phosphoinositides were then extracted, deacylated and analysed by HPLC. Data show levels of glyceroPtdIns3*P* normalised for the levels of glycerophosphatidylinositol (gPtdIns) and expressed as percentage of PtdIns3*P*/gPtdInsP in unstimulated cells (control). Data are means ± SEM from 3 (time points 1 and 5 min) and 1 (time point 10 min) independent experiments.
(TIF)

Table S1 **Primers used for RT-PCR analysis presented in Figure S3.**
(DOC)
