# Supplementary figures and images for: Class II Phosphoinositide 3-Kinases Contribute to Endothelial Cells Morphogenesis
Source: PLoS One. 2013 Jan 8;8(1):e53808. doi: 10.1371/journal.pone.0053808 (PMC3539993; doi:10.1371/journal.pone.0053808)

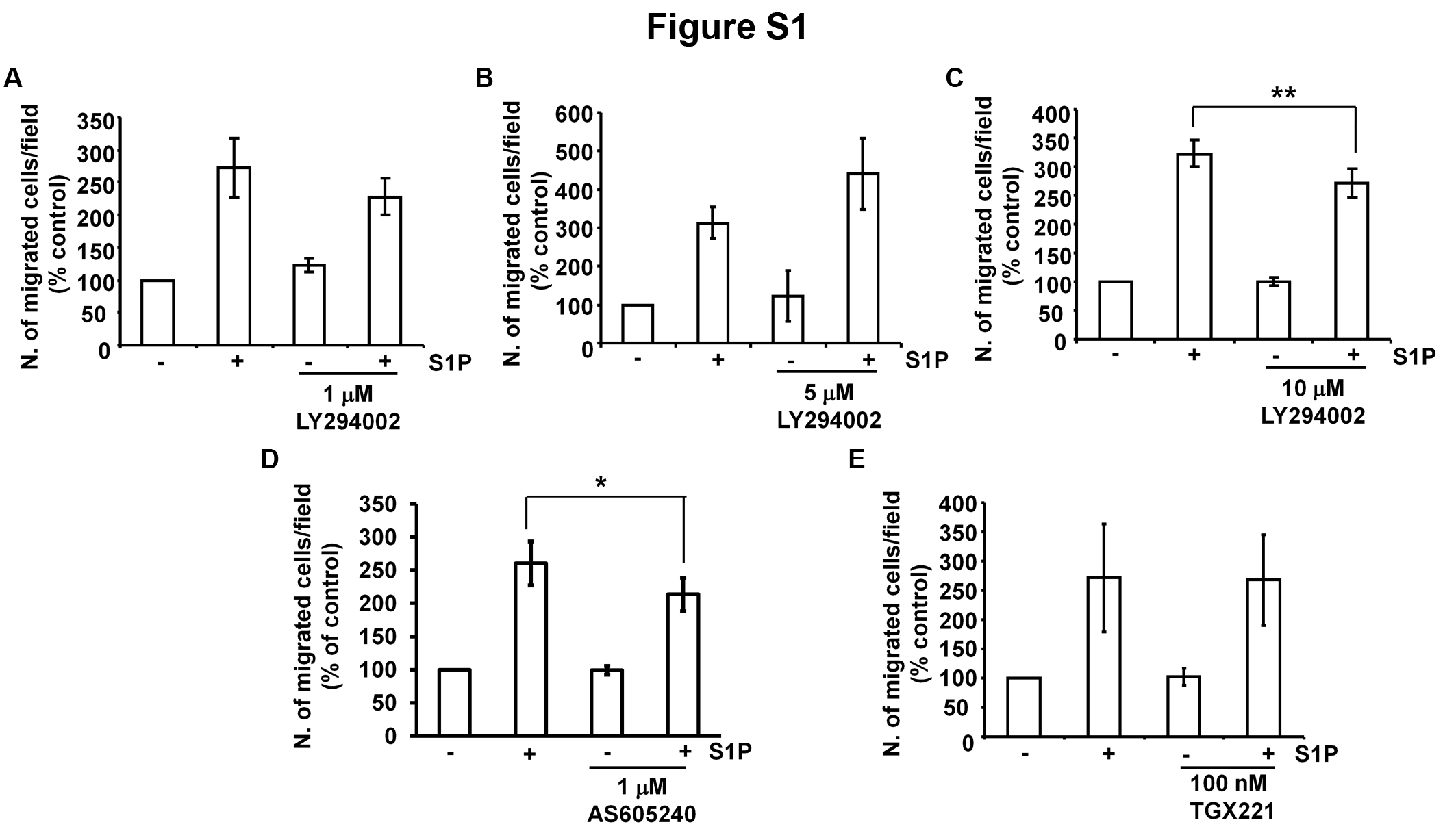

Supplement: Figure S1 — Effect of PI3K inhibitors on S1P- and HDL3-induced EC migration. Serum-starved HUVEC were pre-treated with 1 µM LY294002 (A), 5 µM LY294002 (B), 10 µM LY294002 (C), 1 µM AS605240 (D) or 100 nM of the specific p110β inhibitor TGX221 (D) for 30 min. Cell migration induced by S1P was determined by Transwell assays. Data are expressed as percentage of control (cells untreated and unstimulated) and are means ± SEM from 6 (A), 5 (B), 6 (C), and 3 (D) independent experiments. *p<0.05, **p<0.001. (TIF) [file pone.0053808.s001.tif]

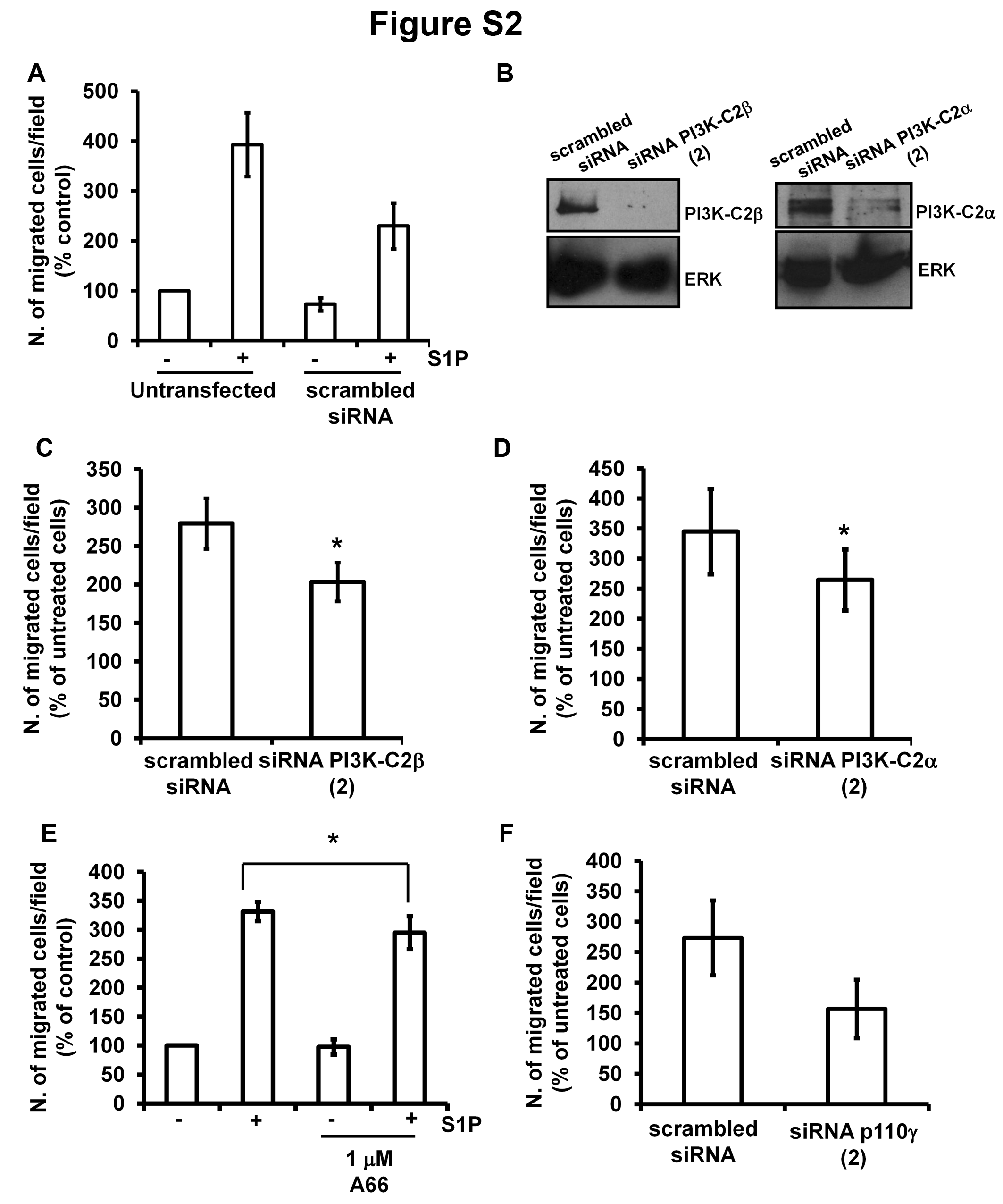

Supplement: Figure S2 — Class II and class IB PI3Ks are involved in S1P-induced EC migration. (A) Results from Transwell assays performed in control, untransfected HUVEC and HUVEC transfected with a scrambled siRNA. Data are expressed as percentage of control (cells untransfected and unstimulated) and are means ± SEM from 6 independent experiments. (B) Levels of PI3K-C2α and PI3K-C2β in cells transfected with specific siRNAs (sequences 2) were assessed by Western blotting. (C-D) Results from Transwell assays performed in HUVEC transfected with the indicated siRNAs. Data are expressed as percentage of each control (cells transfected with each siRNA and unstimulated) and are means ± SEM from 5 (C) and 4 (D) independent experiments.*p<0.05. (E) Results from Transwell assays performed in HUVEC treated with 1 µM of the inhibitor A66. Data are expressed as percentage of control (cells untreated and unstimulated) and are means ± SEM from 4 independent experiments.*p<0.05. (F) Results from Transwell assays performed in HUVEC transfected with a scrambled siRNA or siRNA specifically targeting p110γ (sequence 2). Data are expressed as percentage of each control (cells transfected with each siRNA and unstimulated) and are means ± SEM from 2 independent experiments. (TIF) [file pone.0053808.s002.tif]

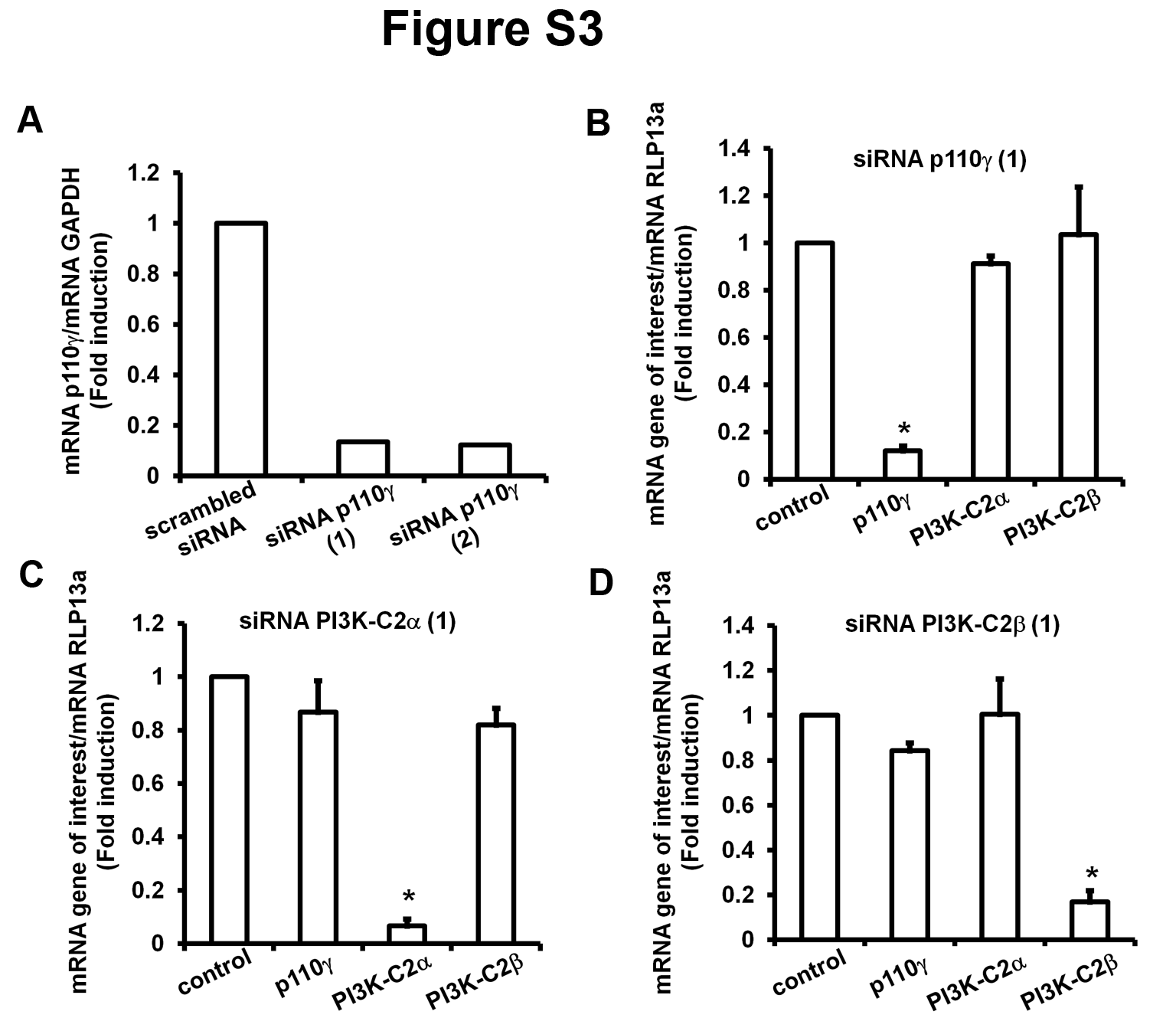

Supplement: Figure S3 — RT-qPCR analysis of PI3Ks levels. (A) Downregulation of p110γ mRNA levels using two distinct siRNAs was determined by RT-qPCR. (B) HUVEC were transfected with siRNAs targeting the indicated PI3Ks. Efficiency and specificity of downregulation was determined by RT-qPCR. (TIF) [file pone.0053808.s003.tif]

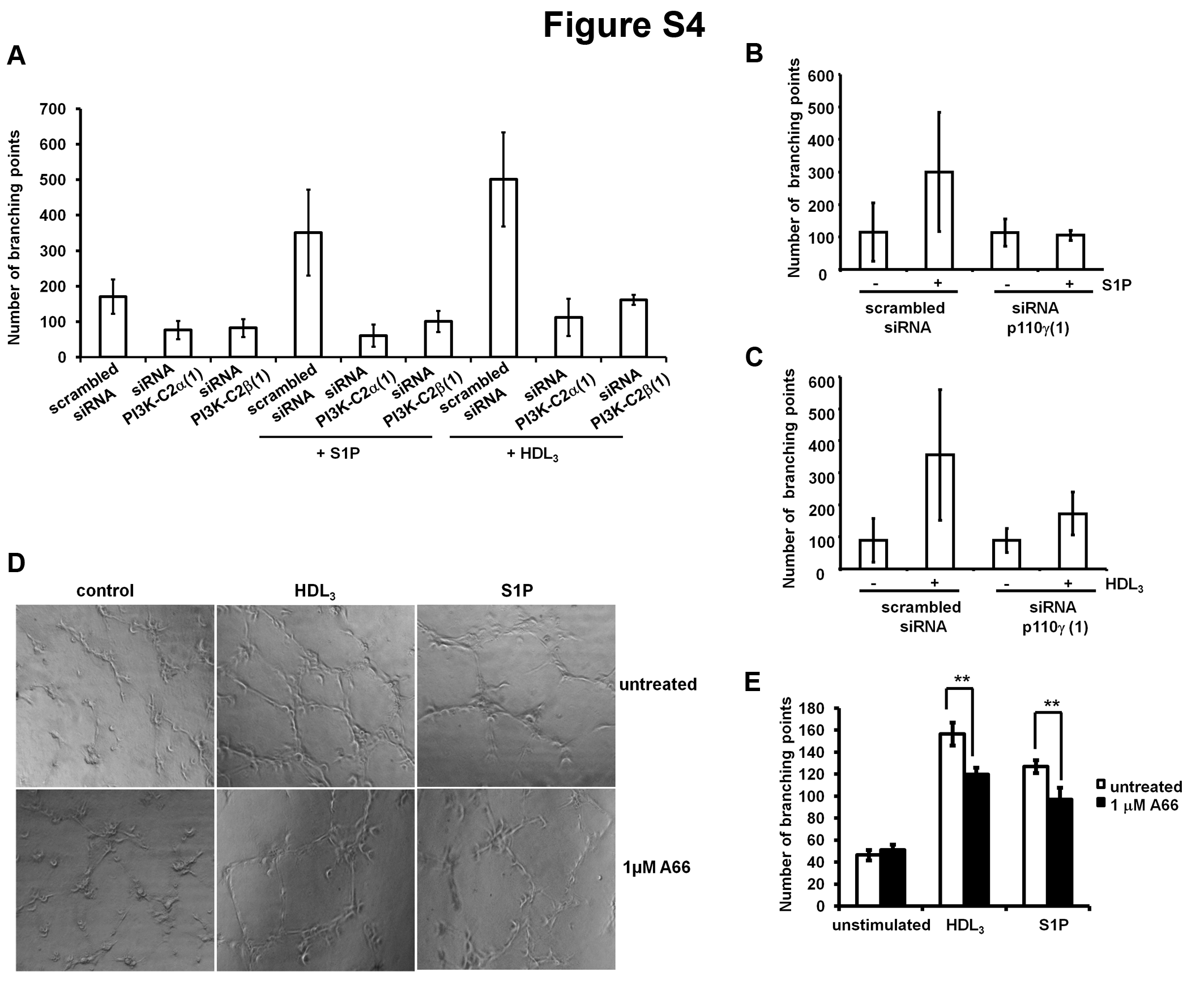

Supplement: Figure S4 — Class II and class IB PI3Ks are involved in remodelling of HUVEC. (A-C) Results from analysis of EC rearrangement. Data indicate the total number of branching points and are means ± SEM from 3–4 (A), 3 (B), and 4 (C) independent experiments. (D,E) The S1P- and HDL3-dependent HUVEC was assessed in the absence or presence of 1 µM A66. Representative images (D) and data indicating the total number of branching points (E) are shown. Data are means ± SEM from 4 independent experiments. **p<0.01. (TIF) [file pone.0053808.s004.tif]

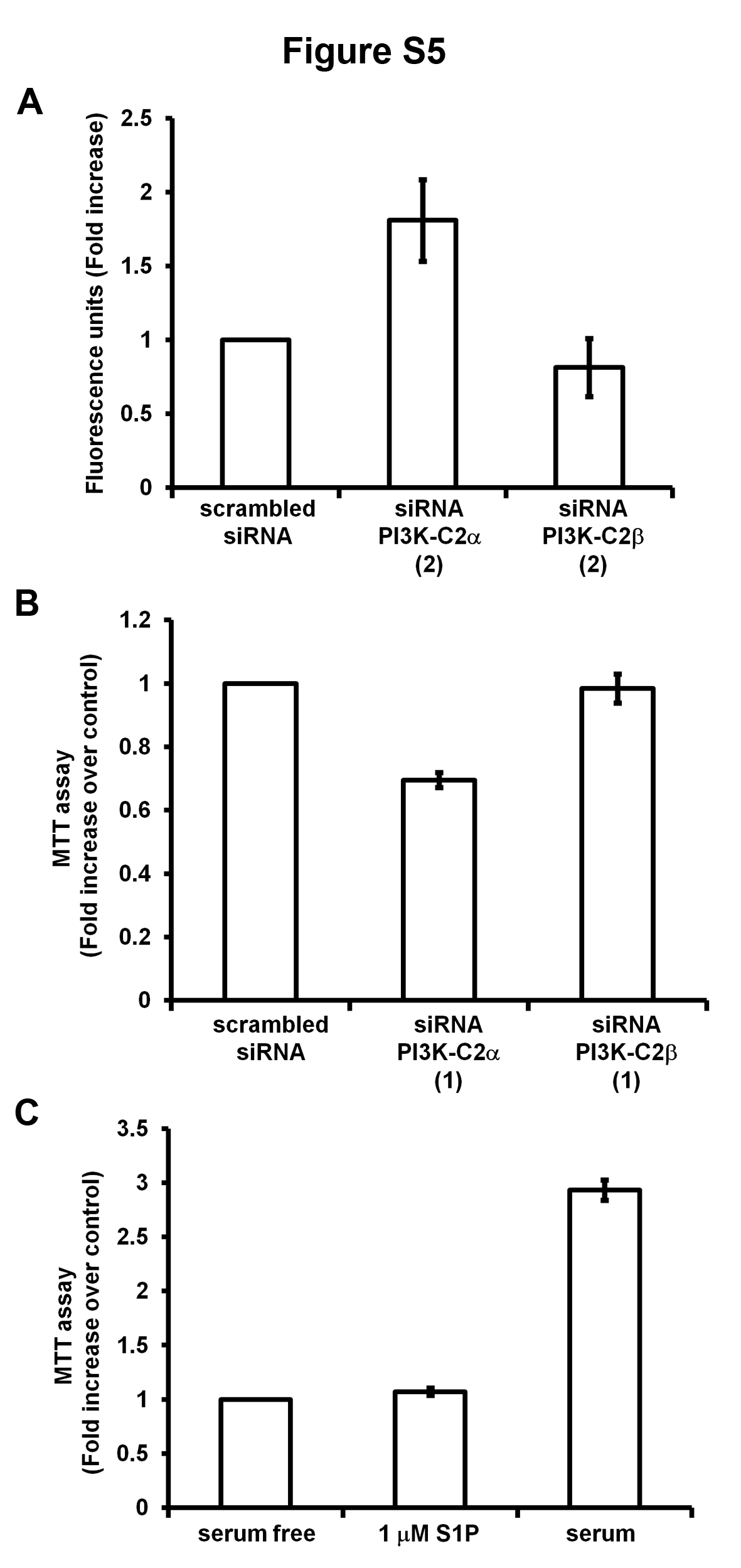

Supplement: Figure S5 — Effect of PI3Ks downregulation on EC apoptosis. (A) Results from caspase 3 assay performed on lysates from HUVEC obtained 48 h after transfection with the indicated siRNAs (sequences 2). Data are means ± SEM from 2 independent experiments. (B) HUVEC transfected with the indicated siRNAs were incubated in serum free M119 after 24 h from transfection. Viability of cells was assessed by MTT assay after further 24 h. Data are expressed as fold increase over control (cells transfected with scrambled siRNA) and are means ± SEM from 4–6 independent experiments. (C) Results from MTT assays performed in HUVEC incubated in serum free M199 or M199 supplemented with 1 µM S1P or 10% FBS. Data are expressed as fold increase over control (cells in serum free media) and are means ± SEM from 5 independent experiments. (TIF) [file pone.0053808.s005.tif]

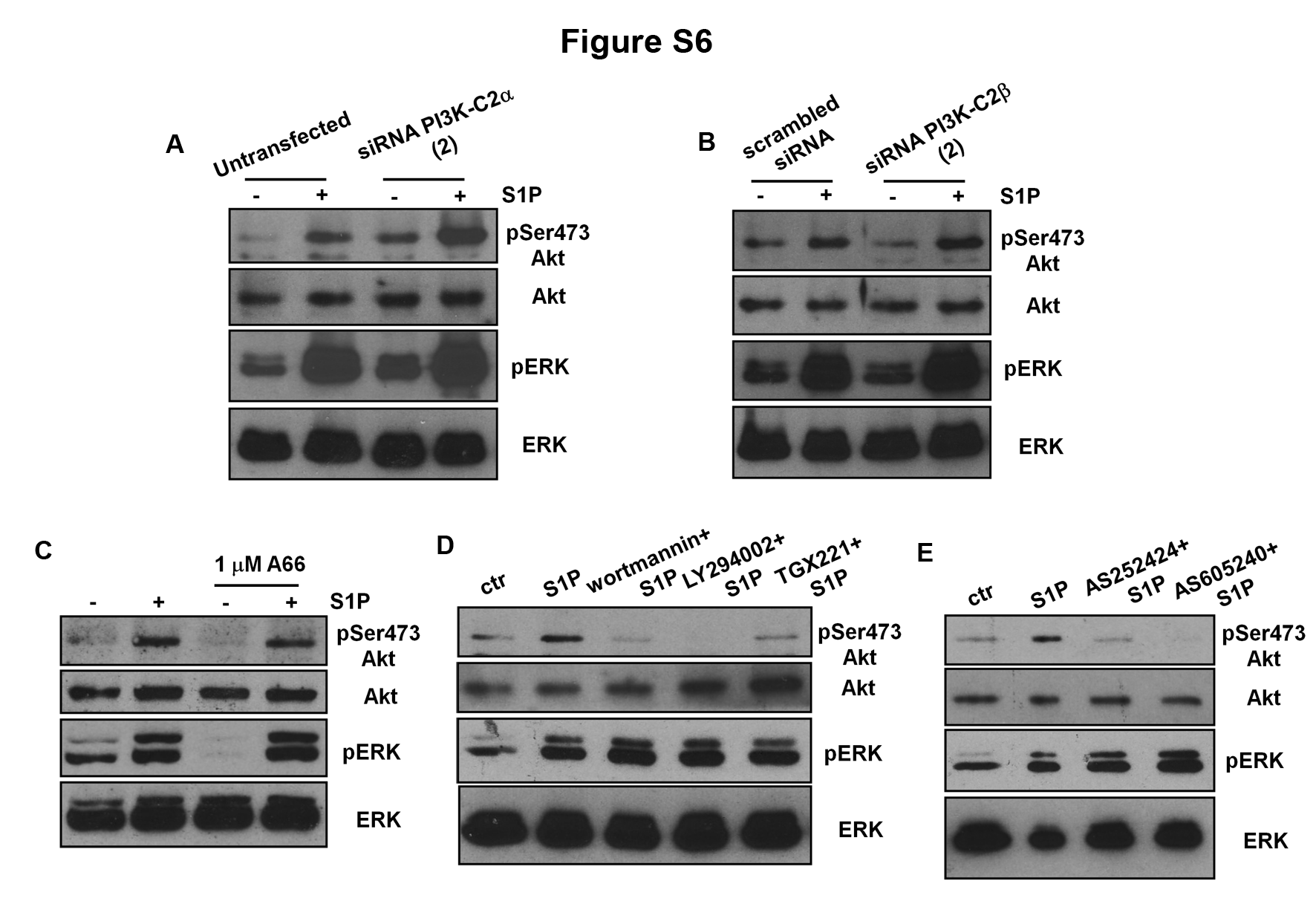

Supplement: Figure S6 — Effect of PI3Ks downregulation and PI3K inhibitors on the S1P-dependent Akt and ERK phosphorylation. Representative images of Western blotting analysis of Akt and ERK phosphorylation performed in HUVEC. Membranes were then stripped and incubated with the corresponding total antibodies. (A-B) HUVEC transfected with the indicated siRNAs were serum starved overnight before stimulation with 1 µM S1P for 10 min. (C) Serum-starved HUVEC were treated with the indicated concentration of A66 for 30 min before stimulation with 1 µM S1P for 10 min in the presence of the inhibitor. (D) Serum-starved HUVEC were treated with 100 nM wortmannin, 10 µM LY294002 or 100 nM TGX221 for 30 min before stimulation with 1 µM S1P for 10 min in the presence of the inhibitors. (E) Serum-starved HUVEC were treated with 1 µM AS252424 or AS605240 for 30 min before stimulation with 1 µM S1P for 10 min in the presence of the inhibitor. (TIF) [file pone.0053808.s006.tif]

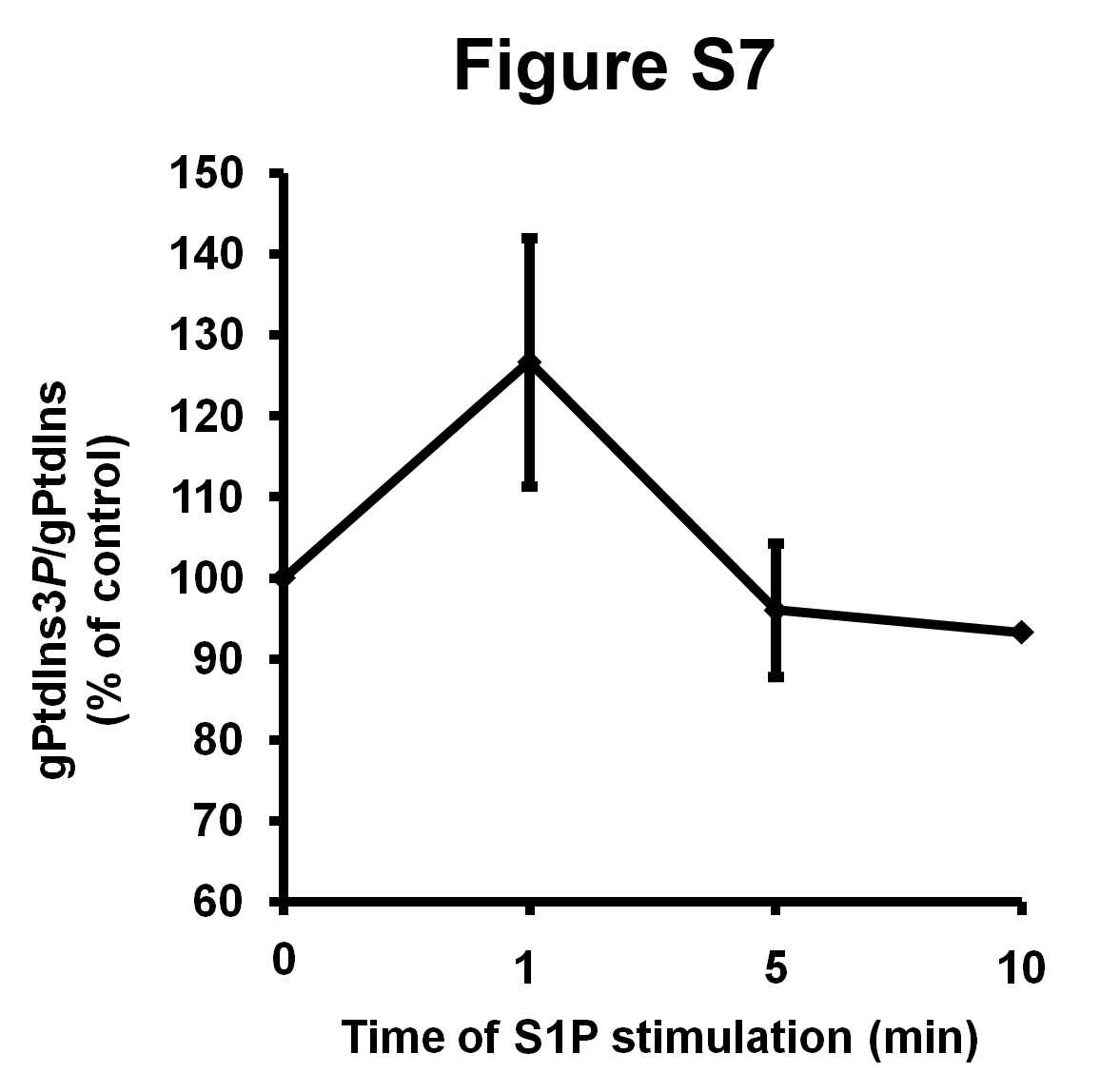

Supplement: Figure S7 — S1P induces de novo synthesis of PtdIns3 P . HUVEC were labelled with [3H]myo-inositol before stimulation with 1 µM S1P for the indicated times. Phosphoinositides were then extracted, deacylated and analysed by HPLC. Data show levels of glyceroPtdIns3P normalised for the levels of glycerophosphatidylinositol (gPtdIns) and expressed as percentage of PtdIns3P/gPtdInsP in unstimulated cells (control). Data are means ± SEM from 3 (time points 1 and 5 min) and 1 (time point 10 min) independent experiments. (TIF) [file pone.0053808.s007.tif]
