# Supplementary material for: Class II Phosphoinositide 3-Kinases Contribute to Endothelial Cells Morphogenesis
Source: PLoS One. 2013 Jan 8;8(1):e53808. doi: 10.1371/journal.pone.0053808 (PMC3539993; doi:10.1371/journal.pone.0053808)
Supplement: Table S1 — Primers used for RT-PCR analysis presented in Figure S3. (DOC) [file pone.0053808.s008.doc]

**Supplementary Table**

|  | **Primer Fw** | **Primer Rev** |
| --- | --- | --- |
| **RLP-13a** | **5’**TAGCTGCCCCACAAAACC**3’** | **3’**TGCCGTCAAACACCCTTGAGA**5’** |
| **p110γ** | **5’**ATCACCGAGACAGGAAACCTATTT**3’** | **5’**CTCTTTATTAATGCCCAGGAAACTTT**3’** |
| **PI3K-C2α** | **5’**ATACCAATCACCGCACAAACC**3’** | **3’**TCTTTGTATACGCCTCTTTTACG**5’** |
| **PI3K-C2β** | **5’**CCTATGCCTCCCGCTATGG**3’** | **3’**GTTCTTGGCGGCCTAAAGA**5** |
